# Reduced anti-Müllerian hormone action in cumulus-oocyte complexes is beneficial for oocyte maturation without affecting oocyte competence

**DOI:** 10.3389/fendo.2024.1365260

**Published:** 2024-06-03

**Authors:** Fuhua Xu, Konstantin Bagnjuk, Nuria Marti-Gutierrez, Sathya Srinivasan, Artur Mayerhofer, David Lee, Tanja Pejovic, Shoukhrat Mitalipov, Jing Xu

**Affiliations:** ^1^ Department of Obstetrics, Gynecology, and Reproductive Sciences, University of Maryland School of Medicine, Baltimore, MD, United States; ^2^ Biomedical Center, Cell Biology, Anatomy III, Faculty of Medicine, Ludwig Maximilian University of Munich, Planegg-Martinsried, Germany; ^3^ Center for Embryonic Cell and Gene Therapy, Oregon Health & Science University, Portland, OR, United States; ^4^ Integrated Pathology Core, Oregon National Primate Research Center, Oregon Health & Science University, Beaverton, OR, United States; ^5^ Department of Obstetrics and Gynecology, School of Medicine, Oregon Health & Science University, Portland, OR, United States; ^6^ Obstetrics and Gynecology Health Center, Providence, Medford, OR, United States; ^7^ Department of Biology & Chemistry, School of Health Sciences, Liberty University, Lynchburg, VA, United States

**Keywords:** anti-Müllerian hormone, cumulus-oocyte complexes, oocyte maturation, oocyte competence, *in vitro* maturation

## Abstract

Anti-Müllerian hormone (AMH) is a key paracrine/autocrine factor regulating folliculogenesis in the postnatal ovary. As antral follicles mature to the preovulatory stage, AMH production tends to be limited to cumulus cells. Therefore, the present study investigated the role of cumulus cell-derived AMH in supporting maturation and competence of the enclosed oocyte. Cumulus-oocyte complexes (COCs) were isolated from antral follicles of rhesus macaque ovaries for *in vitro* maturation with or without AMH depletion. Oocyte meiotic status and embryo cleavage after *in vitro* fertilization were assessed. *In vitro* maturation with AMH depletion was also performed using COCs from antral follicles of human ovarian tissue. Oocyte maturation and morphology were evaluated. The direct AMH action on mural granulosa cells of the preovulatory follicle was further assessed using human granulosa cells cultured with or without AMH supplementation. More macaque COCs produced metaphase II oocytes with AMH depletion than those of the control culture. However, preimplantation embryonic development after *in vitro* fertilization was comparable between oocytes derived from COCs cultured with AMH depletion and controls. Oocytes resumed meiosis in human COCs cultured with AMH depletion and exhibited a typical spindle structure. The confluency and cell number decreased in granulosa cells cultured with AMH supplementation relative to the control culture. AMH treatment did not induce cell death in cultured human granulosa cells. Data suggest that reduced AMH action in COCs could be beneficial for oocyte maturation. Cumulus cell-derived AMH is not essential for supporting oocyte competence or mural granulosa cell viability.

## 1 Introduction

Anti-Müllerian hormone (AMH) has been considered as a key paracrine/autocrine factor regulating folliculogenesis in the postnatal ovary. In mice, AMH inhibits primordial follicle recruitment into the growing pool and the cyclic recruitment of follicle-stimulating hormone (FSH)-sensitive follicles (1). In primate species, after primordial follicle activation, AMH is produced by granulosa cells (GCs) of growing follicles (2). AMH production levels increase during the preantral stage of follicular development, which is suggested to regulate GC proliferation and maintain oocyte viability (3). During antrum formation, GCs differentiate into mural GCs lining the follicle wall and cumulus cells (CCs) surrounding the oocyte. As the antral follicle continues to grow, AMH production declines in mural GCs, which contributes to the decreased AMH concentrations in follicular fluid of large antral follicles (4). Because AMH inhibits estradiol biosynthesis, diminished AMH level is critical for antral follicle maturation (3). In preovulatory follicles, AMH production tends to be limited to CCs (5). Since AMH-specific type II receptor is expressed in the oocyte of preovulatory follicle (6), research has been conducted to determine effects of AMH retained in CCs on the enclosed oocyte; but results remain inconclusive.

The direct actions of AMH on oocyte maturation and competence are often investigated during the *in vitro* maturation (IVM) process. An early study in rats suggested that AMH supplementation in cumulus-oocyte complex (COC) culture inhibited oocyte maturation by preventing the resumption of meiosis (7). Similar inhibitory effect was also observed in mouse IVM, during which AMH attenuated FSH-induced cumulus expansion and oocyte maturation (8). However, inconsistent results were reported from other research groups. Although oocyte maturation was not affected, AMH supplementation during IVM improved oocyte competence in mice with an increased blastocyst formation rate after *in vitro* fertilization (IVF) (9). In addition, AMH promoted oocyte maturation when COCs obtained from patients undergoing controlled ovarian stimulation were cultured (6). Furthermore, in some studies, AMH effects were not evident. For example, neither an inhibitory nor a stimulatory action on meiosis resumption was significant when rat oocytes were treated with AMH *in vitro* (10). AMH did not produce any significant effects on the preimplantation development of embryos derived from beef and dairy cattle COCs following IVM with AMH supplementation (11). The inconsistency of current data could be due to differences in health conditions of animals and patients, ovarian stimulation protocols to generate antral follicles, as well as AMH protein sources and doses used for IVM. Thus, additional investigations are warranted to discern the role of AMH in the COC of antral follicles.

IVM has a potential application in fertility preservation. For patients who are not suitable for ovarian stimulation, e.g., prepubertal and adolescent girls, IVM of COCs isolated from antral follicles in the ovarian tissue can produce mature oocytes for cryopreservation (12). Therefore, in the present study, the role of AMH in supporting oocyte maturation and competence was determined by AMH depletion during IVM. COCs were collected from healthy rhesus macaques, a nonhuman primate model with similar ovary biology and physiology to those of humans, without experiencing ovarian stimulation. IVM was also performed using COCs obtained from unstimulated human ovarian tissue of pediatric patients. The direct AMH action on mural GCs in large antral follicles was further assessed using human GC culture. According to AMH expression pattern during primate folliculogenesis, it was hypothesized that diminished AMH production is necessary to sustain oocyte development and GC viability in antral follicles.

## 2 Materials and methods

### 2.1 Ethical approval

The care of rhesus macaques (*Macaca mulatta*) was provided by the Division of Comparative Medicine, Oregon National Primate Research Center (ONPRC), Oregon Health & Science University (OHSU). Animals were treated according to the National Institutes of Health (NIH)’s Guide for the Care and Use of Laboratory Animals. Protocols were approved by the ONPRC Institutional Animal Care and Use Committee (13).

Human ovarian tissue and clinical information were obtained from the Oregon Ovarian Cancer Registry and Tissue Repository at OHSU. Ovarian tissue was collected with written informed consent and de-identified. The experimental protocol was approved by the Institutional Review Board (#921) at OHSU (14).

Collection of human IVF-derived GCs was approved by the ethics committee of Ludwig-Maximilians-University in Munich. Written informed consents were obtained from patients (15).

### 2.2 IVM of nonhuman primate COCs

Eight female macaques (8–10 year old) were assigned to the study. Hemiovariectomies were conducted by laparoscopy during day 1–4 of the menstrual cycles, i.e., early follicular phase. Ovaries were kept in the Holding Media (Cooper Surgical, Inc.) at 37°C. COCs were obtained by puncturing small antral follicles (0.5–2 mm) with a needle and cultured individually for 48 hours at 37°C and 20% O_2_/5% CO_2_/75% N_2_ in 100 µl Tyrode’s albumin lactate pyruvate media containing 5% (v/v) monkey serum, 75 mIU/ml recombinant human FSH (Merck & Co.), 75 mIU/ml recombinant human luteinizing hormone (LH; EMD Serono), and 10 ng/ml amphiregulin (MilliporeSigma), as previously described (16). Because CCs of antral follicles produce appreciable levels of AMH (5), the role of endogenous AMH in supporting oocyte maturation and competence was examined by AMH depletion. Experimental groups included (a) media-only control (n = 37; 5–16 COCs/animal) and (b) 100 ng/ml neutralizing anti-human AMH antibody (AMH-Ab; MAB1737; R&D Systems, Inc.) (n = 38; 6–16 COCs/animal). After IVM, conventional IVF and the subsequent embryo culture were performed as previously reported (13). Briefly, oocytes were denuded mechanically by gentle pipetting to assess meiotic status. A germinal vesicle-intact (GV) oocyte contains a large nucleus covered by a nuclear envelope. After germinal vesicle breakdown, nuclear envelope becomes invisible in a metaphase I (MI) oocyte. The presence of the first polar body indicates oocyte maturation to the metaphase II (MII) stage. GV and MI oocytes were counted before being discarded, whereas MII oocytes were maintained in Tyrode’s albumin lactate pyruvate media at 37°C and 20% O_2_/5% CO_2_/75% N_2_ for IVF as previously described (17). Macaque semen was provided by the Assisted Reproductive Technologies Core at ONPRC, OHSU. The resulting zygotes were transferred to 100 μl Global Medium (LifeGlobal Group) and cultured at 37°C and 5% O_2_/6% CO_2_/89% N_2_. Embryonic development was evaluated by microscopy and embryo cleavage was recorded.

### 2.3 IVM of human COCs

Patients (11–14 years old; n = 3) underwent oophorectomy due to ovarian tumors or prophylactic treatment (18) (**Table 1**). The patients’ menstrual cycle stages were unknown. Ovarian tissue was collected intraoperatively and kept in the Holding Media (Cooper Surgical, Inc.) at 37°C. COCs were obtained by puncturing all visible small antral follicles (2–4 mm) with a needle for IVM as previously reported (14). Briefly, COCs (n = 24; 7–10 COCs/patient) were cultured individually for 48 hours at 37°C and 5% O_2_/5% CO_2_/90% N_2_ in 100 μl SAGE *In-Vitro* Maturation Media (CooperSurgical) supplemented with 75 mIU/ml recombinant human FSH and 75 mIU/ml recombinant human LH, and 100 ng/ml neutralizing anti-human AMH-Ab to deplete endogenous AMH produced by CCs. After IVM, oocytes were evaluated as previously reported (14). Briefly, oocytes were denuded to assess meiotic status and diameter. The meiotic spindle of MII oocytes was visualized by polarized microscopy using an Oosight Imaging System (Cambridge Research & Instrumentation).

**Table 1:** Patients enrolled and *in vitro* maturation outcomes.

	Patient 1	Patient 2	Patient 3
Age (year)	11	13	14
Race/Ethnicity	White/Hispanic	White/Non-Hispanic	White/Non-Hispanic
Diagnosis	Teratoma	*SMARCA4* mutation	Androblastoma
Surgery	Unilateral oophorectomy	Bilateral oophorectomy	Unilateral oophorectomy
Cumulus-oocyte complexes (n)	7	7	10
Germinal vesicle oocytes (n)	4	4	2
Metaphase I oocytes (n)	0	1	1
Metaphase II oocytes (n)	3	2	7
Metaphase oocyte diameter (μm)*	117.1 ± 0.8	118.9 ± 2.1	116.1 ± 1.3

Values are mean ± SEM with each oocyte as an individual data point. SMARCA4, SWI/SNF-related matrix-associated actin-dependent regulator of chromatin subfamily A member 4.

While GV and MI oocytes were discarded after counting, to further assess oocyte spindle structure, MII oocytes were fixed in 4% paraformaldehyde (Electron Microscopy Sciences) for immunofluorescence staining as previously described (19). Briefly, oocytes were incubated with primary antibody overnight at 4°C and secondary antibody for 1 hour at room temperature. Spindle microtubules were labeled with α-tubulin clone DM1A (1:100; MABT205; MilliporeSigma) followed by Alexa Fluor 488 goat anti-mouse IgG (1:500; A-11001; Thermo Fisher Scientific). Chromosomes were labeled with 5 μM ethidium homodimer (Thermo Fisher Scientific). Spindle images were captured by a Leica SP5 AOBS confocal microscope (Leica Microsystems). The objective PL APO CS 63× 1.3 GLY UV was used to collect Z-stack data sets with images taken every 0.5 µm.

### 2.4 Culture of human GCs

Human GC isolation from follicular fluid and cell culture were performed as previously described (15). Briefly, 10^5^ cells were seeded per 35 mm^2^ plate and cultured for 72 hours at 37 °C and 5% CO_2_ in DMEM/F12 media supplemented with 10% (v/v) fetal calf serum, 100 U/ml penicillin, and 100 µg/ml streptomycin. Because AMH production by GCs of large antral follicles is barely detectable, if not absent (20), the direct action of AMH on isolated GCs was examined by AMH supplementation in cell culture. Experimental groups included (a) media-only control and (b) 50 ng/ml recombinant human AMH protein (rhAMH; 1737-MS; R&D Systems, Inc.) supplementation. AMH dosage was chosen based on a previous dose-response study using human GC culture supplemented with rhAMH of the same source (21). Confluency of cultured GCs (n = 4 patients) was measured using the JuLI Br Bright-cell Movie Analyzer (NanoEntek). Confluency data were collected every 20 min and normalized to the corresponding control. Cell counting (n = 7 patients) was conducted using the Cell Counter & Analyzer CASY (OMNI Life Science GmbH & Co KG).

Protein isolation and Western Blot were conducted as previously described (15). Briefly, cultured GCs were lysed for total protein extraction using a radioimmunoprecipitation buffer with protease/phosphatase inhibitors (Thermo Fisher Scientific) and subsequent 12% SDS-PAGE. After blotting, the membrane was blocked with 5% nonfat milk in Tris-buffered saline with Tween 20 (pH 7.4). Apoptosis and necroptosis were evaluated using an Apoptosis/Necroptosis Antibody Sampler Kit (92570; Cell Signaling Technology) including primary antibodies anti-MLKL (mixed lineage kinase domain-like pseudokinase), phospho-MLKL, caspase-3, cleaved caspase-3, caspase-8, and cleaved caspase 8, as well as HRP-conjugated anti-rabbit and anti-mouse secondary antibodies. Anti-β-actin antibody (A5441; MilliporeSigma) was used as a loading control. Autophagy was detected using an CYTO-ID Autophagy detection kit (ENZ-51031–0050; Enzo Life Sciences, Inc.) according to the manufacturer’s protocol. Images of autophagosomes stained with Cyto-ID Green autophagy dye and nuclei stained with Hoechst 33342 were captured by fluorescence microscopy (22). Fluorescence intensity relative to the cell size was analyzed, with background subtracted, using an image processing and analysis package in Java (Fiji, ImageJ).

### 2.5 Statistical analyses

Statistical analysis was performed using SAS software (SAS Institute Inc). Data represent 8 animals in macaque IVM, 3–8 metaphase oocytes from human IVM, as well as 4 and 7 patients for the GC confluency assay and cell counting, respectively. Because COCs from each animal were randomly distributed into the control and AMH-Ab treatment groups, Wilcoxon signed-rank test was used to evaluate differences in oocyte maturation and embryo cleavage rates with each animal as a unit. Kruskal-Wallis test was used to compare oocyte diameters with each oocyte as an individual data point. Paired Student’s t-test was used to analyze GC confluency and cell counting data. Differences were considered significant at P < 0.05 and values are presented as mean ± SEM.

## 3 Results

### 3.1 IVM of nonhuman primate COCs

**Figure 1:**
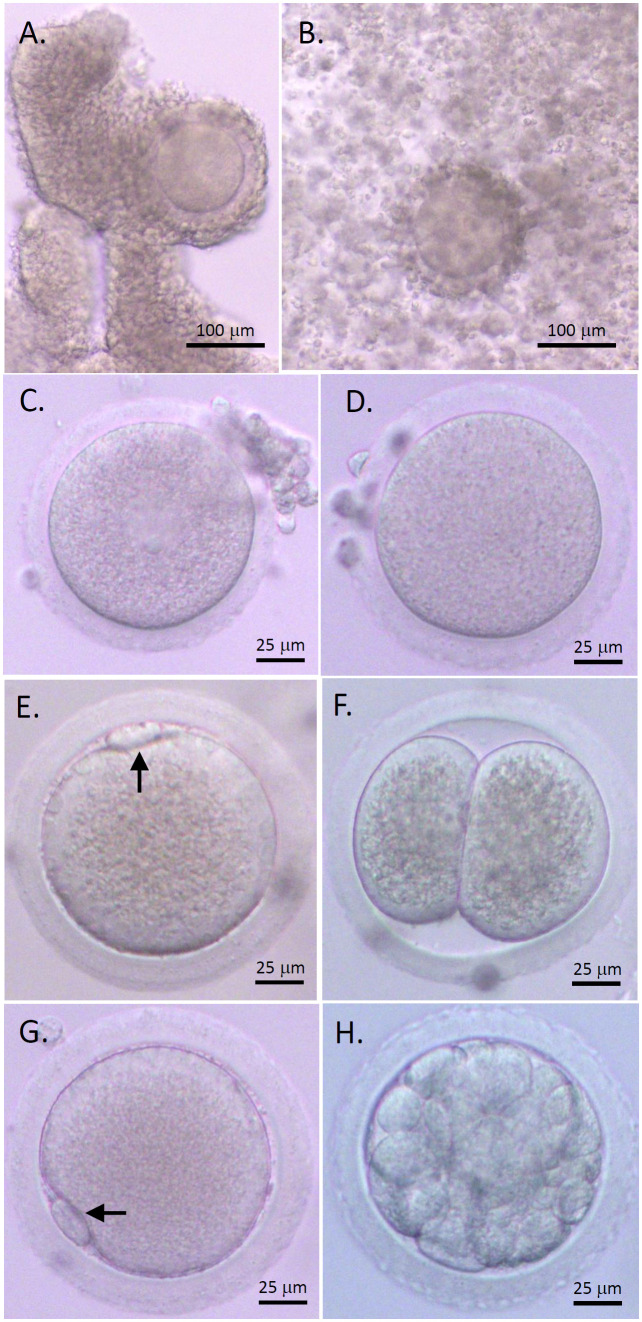
Rhesus macaque oocyte maturation and competence after *in vitro* maturation (IVM) with anti-Müllerian hormone (AMH) depletion. Cumulus-oocyte complexes (COCs) underwent IVM with neutralizing anti-human AMH antibody addition **(A)** Cumulus expansion was observed 48 hours post-IVM **(B)** Healthy germinal vesicle-intact **(C)**, metaphase I **(D)**, and metaphase II (MII; **E, G**) oocytes were obtained. The first polar body (arrows) was observed with normal size in MII oocytes **(E, G)**. Following *in vitro* fertilization (IVF), the first cleavage occurred within 24 hours to generate 2-cell embryos **(F)** Preimplantation embryos developed to the morula stage within 5 days post-IVF **(H)**. Scale bar = 100 μm for COCs and 25 μm for oocytes and embryos.

COCs isolated from macaque antral follicles consisted of a healthy GV oocyte, without vacuoles or condensed cytoplasm, surrounded by layers of CCs (**Figure 1A**). Following 48 hours of IVM, cumulus expansion was observed in all cultured COCs of the control and AMH-Ab-treated groups (**Figure 1B**). All oocytes exhibited typical morphology after IVM including GV oocytes without resuming meiotic division (**Figure 1C**), MI oocytes experienced GV breakdown (**Figure 1D**), and MII oocytes extruding the first polar body (**Figures 1E, G**). Less than 50% of MII oocytes were fertilized (**Table 2**) with the first cell cleavage occurring within 24 hours post-IVF to become 2-cell embryos (**Figure 1F**). Preimplantation embryos developed to the morula stage within 5 days post-IVF (**Figure 1H**).

**Table 2:** *In vitro* maturation of rhesus macaque oocytes.

	Control	AMH-Ab
Germinal vesicle oocytes (%)	62.8 ± 13.0	36.4 ± 12.6
Metaphase I oocytes (%)	15.3 ± 9.8	20.0 ± 7.1
Metaphase II oocytes (%)	21.9 ± 10.4	43.6 ± 8.5*
Cleaved embryos (%)	33.3 ± 23.6	43.8 ± 19.1

Values are mean ± SEM with each animal as an individual data point. *, significant difference between culture groups (P < 0.05). AMH-Ab, anti-Müllerian hormone antibody treatment.

The percentages of GV and MI oocytes obtained after IVM were comparable between the control and AMH-Ab-treated COCs. More (P < 0.05) COCs produced MII oocytes with AMH-Ab addition than those of the control culture (**Table 2**). However, there were no significant differences between MII oocytes derived from the control and AMH-Ab-treated COCs in terms of embryo cleavage rates post-IVF (**Table 2**).

### 3.2 IVM of human COCs

**Figure 2:**
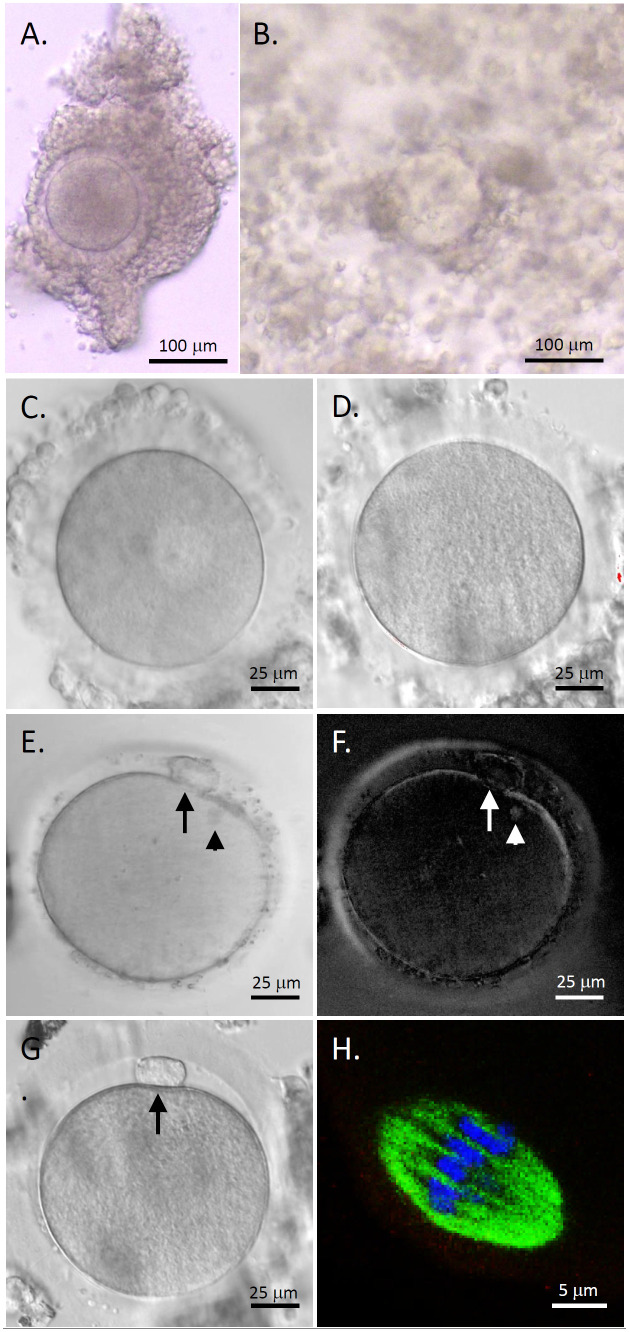
Human oocyte maturation and morphology after *in vitro* maturation (IVM) with anti-Müllerian hormone (AMH) depletion. Cumulus-oocyte complexes (COCs) underwent IVM with neutralizing anti-human AMH antibody addition **(A)** Cumulus expansion was observed 48 hours post-IVM **(B)** Healthy germinal vesicle-intact **(C)**, metaphase I **(D)**, and metaphase II (MII; **E, G**) oocytes were obtained. The first polar body (arrows) and meiotic spindle (arrowheads) were observed with normal sizes and positions in MII oocytes by polarized microscopy **(E, F)**. Immunofluorescence (green, tubulin; blue, DNA) shows a spindle with properly aligned chromosomes in MII oocytes **(H)** Scale bar = 100 μm for COCs, 25 μm for oocytes, and 5 μm for the spindle.

COCs isolated from human antral follicles consisted of a healthy GV oocyte, without vacuoles or condensed cytoplasm, surrounded by layers of CCs (**Figure 2A**). Following 48 hours of IVM, cumulus expansion was observed in all cultured COCs (**Figure 2B**). All oocytes exhibited typical morphology after IVM including GV (**Figure 2C**), MI (**Figure 2D**), and MII (**Figures 2E, G**) oocytes. The first polar body and meiotic spindle were observed with normal sizes and positions in MII oocytes, as indicated by polarized microscopy (**Figures 2E, F**) (14). Confocal microscopy imaging showed a typical barrel-shaped spindle with properly aligned chromosomes (**Figure 2H**) (16).

As summarized in **Table 1**, a total of 24 COCs (8 ± 1 COCs/patient) harvested from the ovarian tissue of three pediatric patients underwent IVM with AMH-Ab addition. While (44.8 ± 12.4)% of the oocytes remained at the GV stage, (55.2 ± 12.4)% resumed meiosis and (47.1 ± 12.1)% matured to the MII stage. Diameters of all MII oocytes were greater than 110 µm. There were no significant differences in diameters of MII oocytes between patients (**Table 1**).

### 3.3 Culture of human GCs

**Figure 3:**
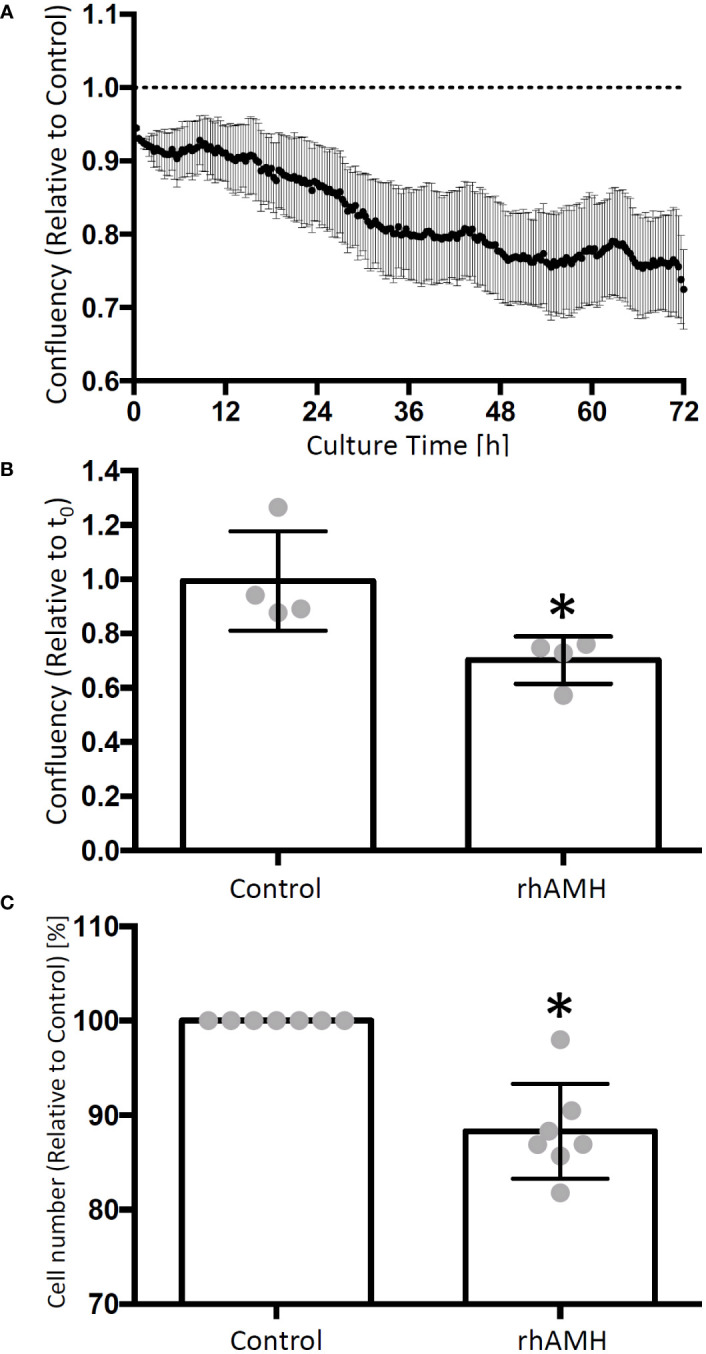
Effects of anti-Müllerian hormone (AMH) on human granulosa cell (GC) growth in culture. The cell confluency data were collected every 20 min and normalized to the corresponding control. GC confluency decreased over time in culture supplemented with recombinant human AMH (rhAMH) relative to the control culture **(A)** At the end of culture, cell confluency was lower for rhAMH-treated GCs than that of the control GCs **(B)** Cell numbers decreased for GCs treated with rhAMH relative to controls **(C)** *, significant difference between culture groups, P < 0.05. Data are presented as mean ± SEM.

GC confluency decreased over time in culture supplemented with rhAMH relative to the control culture (**Figure 3A**). At the end of culture, the confluency was significantly lower (P < 0.05) for rhAMH-treated GCs than that of the control GCs (**Figure 3B**). Cell numbers also decreased (P < 0.05) for GCs treated with rhAMH relative to controls (**Figure 3C**).

**Figure 4:**
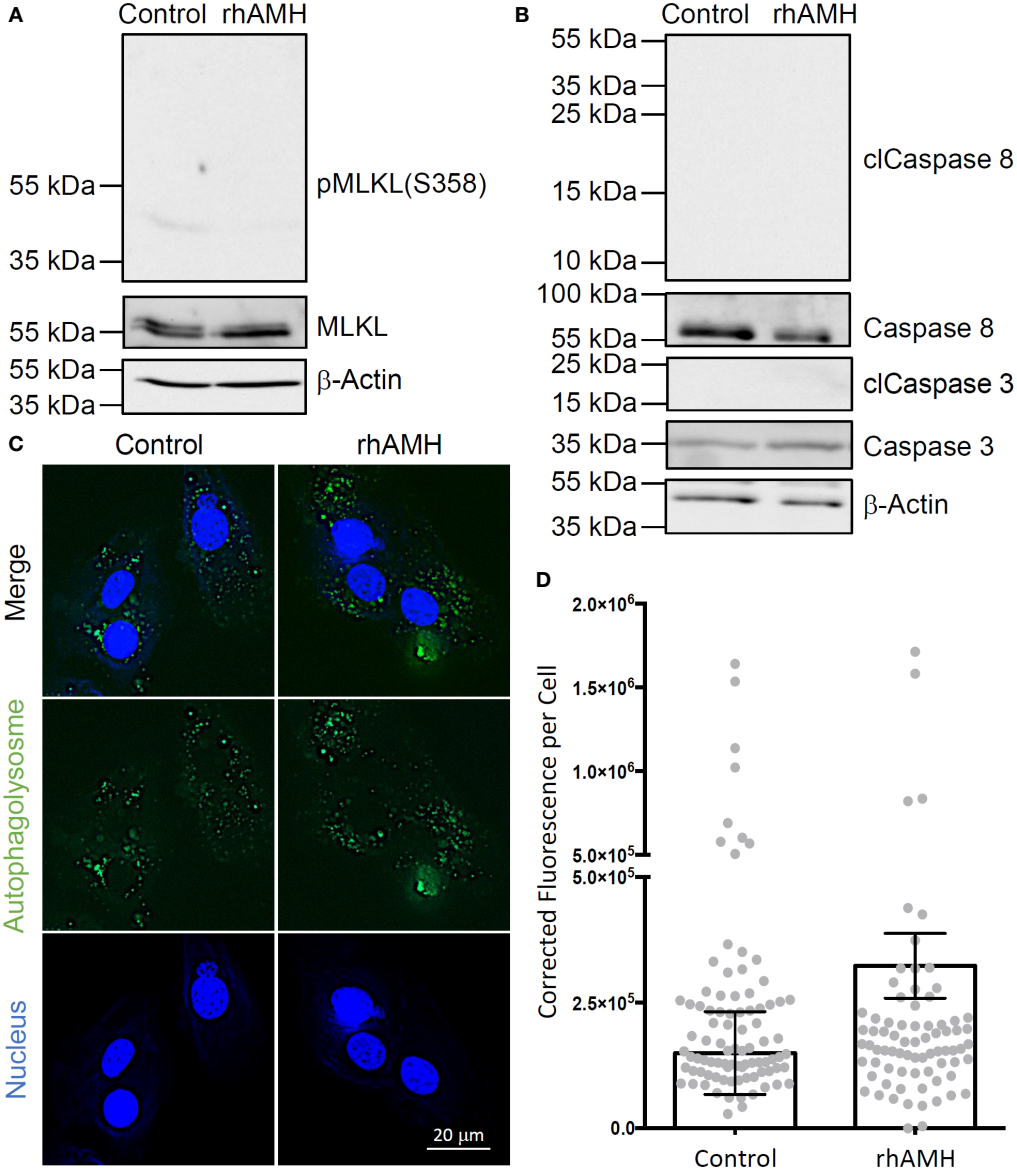
Effects of anti-Müllerian hormone (AMH) on programmed cell death of human granulosa cells (GCs) in culture. While mixed lineage kinase domain-like pseudokinase (MLKL; ~55 kDa) was detected in the control and recombinant human AMH (rhAMH)-treated GCs by Western blot, phospho-MLKL (necroptosis indicator; 35–55 kDa) signal was minimal **(A)**. While uncleaved caspase 3 (~35 kDa) and caspase 8 (~55 kDa) were detected, cleaved protein fragments (apoptosis indicators; clCaspase 3, 15–25 kDa; clCaspase 8, 10–55 kDa) were not detected in the control or rhAMH-treated GCs by Western blot **(B)**. The β-actin protein served as the loading control **(A, B)**. Fluorescence microscopy shows autophagolysosomes (green) in both the control and rhAMH-treated GCs (blue, DNA) **(C)**. Fluorescence intensity was slightly, but not significantly, elevated in GCs treated with rhAMH relative to controls **(D)**.

A ~55 kDa protein band was identified in all GC samples, which is consistent with the predicted band size of human MLKL protein (**Figure 4A**). Phospho-MLKL signal between 35 kDa and 55 kDa was very faint and did not exhibit significant differences between the control GCs and GCs treated with rhAMH (**Figure 4A**). A ~35 kDa and a ~55 kDa protein bands were identified in all GC samples, which are consistent with the predicted band sizes of uncleaved caspase 3 and caspase 8 proteins, respectively (**Figure 4B**). Cleaved caspase 3 and caspase 8 fragments were not detected between 15–25 kDa or 10–55 kDa, respectively, in the control GCs or GCs treated with rhAMH (**Figure 4B**). Autophagolysosomes were visible in both the control and rhAMH-treated GCs (**Figure 4C**). Fluorescence intensity was slightly, but not significantly, elevated in GCs treated with rhAMH relative to controls (**Figure 4D**).

## 4 Discussion

Since AMH protein expression is retained in CCs during antral follicle maturation, the present study investigated the potential effect of this paracrine factor on the enclosed oocyte. For the first time, the role of AMH in supporting oocyte development in COCs was determined in primate species utilizing IVM. Data suggest that AMH production by CCs may negatively impact oocyte nuclear maturation, although it does not diminish oocyte competence in the subsequent preimplantation embryonic development following IVF. It also appears that AMH negatively impacts the viability of mural GCs in antral follicles.

To preclude the influence from mural GCs, COCs were isolated from macaque antral follicles for IVM. AMH depletion was achieved using a neutralizing AMH-Ab known for effectively blocking endogenous AMH signaling in nonhuman primate follicles (13). The dampened endogenous AMH action appeared to be beneficial to oocyte maturation *in vitro* by doubling the percentages of MII oocytes derived from IVM. However, oocyte quality was not affected by AMH depletion as determined by embryo cleavage rates after IVF. Data are consistent with previous studies in other animal models. *In vitro* and *in vivo* evidence in rats suggested an inhibitory role of AMH in oocyte maturation in COCs of antral follicles. When COCs isolated from unstimulated ovaries underwent IVM, AMH supplementation in the media prevented meiosis resumption and GV breakdown which was cyclic AMP-dependent (7). During spontaneous estrous cycle, AMH production in CCs of antral follicles decreased prior to ovulation (23). The diminished AMH protein expression in CCs was also observed when rats were treated with gonadotropin to induce ovulation (23). In a study using dairy and beef cattle without experiencing ovarian stimulation, COCs were isolated from ovaries for IVM (11). Although oocyte maturation was not assessed, embryo cleavage rates and percentages of blastocysts obtained post-IVF were comparable between the control and rhAMH-treated COCs. It appears that, while not affecting oocyte competence, decreased AMH expression in CCs of antral follicles could be beneficial to oocyte nuclear maturation. Notably, ovarian stimulation may affect the response of isolated COCs to AMH treatment *in vitro*. For example, oocyte maturation rates were not altered by AMH supplementation during IVM, when COCs were obtained from rat (10) or mouse (8, 9) ovaries primed by gonadotropins. It could be due to the down-regulated AMH-specific type II receptor expression in COCs of antral follicles by gonadotropic and steroid hormones (24). Nevertheless, AMH production by CCs of antral follicles does not appear to be crucial for oocyte maturation in animal models.

The essentialness of CC-derived AMH for oocyte maturation was further tested in human IVM. AMH depletion did not prevent oocyte meiotic division or alter oocyte morphology. Although, owing to the high heterogeneity of patients and lack of a control group, the beneficial effects of AMH depletion remained undetermined, oocyte maturation rates in the present study were higher compared with data from previous studies in pediatric patients (10.3–32% MII oocytes) using similar control IVM conditions (12, 25, 26). Currently, most of the clinical studies are conducted in patients seeking infertility treatment, such as IVF/intracytoplasmic sperm injection (ICSI) and IVM. Data support the association between reduced levels of CC-derived AMH and improved oocyte development. For example, levels of *AMH* mRNA were higher in CCs from compacted COCs than those of expanded COCs after IVM (27). In patients undergoing ovarian stimulation, AMH protein expression in CCs decreased when antral follicles matured to the preovulatory stage (27). In preovulatory follicles, *AMH* mRNA levels were lower in CCs of those containing MII oocytes than those containing GV, MI or atretic oocytes (28). When MII oocytes were assessed for morphological quality before ICSI, *AMH* mRNA levels in CCs surrounding the morphologically optimal oocytes were lower than those of CCs surrounding oocytes with suboptimal morphology (29). However, levels of CC-derived AMH were not correlated with the morphological quality of zygotes and embryos resulted from ICSI or the embryo transfer outcomes (29). Interestingly, *AMH* mRNA levels in CCs of the preovulatory follicle increased in patients with advanced reproductive age (40–45 years) compared with those of younger patients (21–35 years), indicating a possible involvement of CC-derived AMH in reproductive aging (5). It appears that an intensive AMH expression by CCs of antral follicles is not essential for oocyte maturation or competence. Recently, a human IVM study reported a stimulatory effect of AMH supplementation on oocyte maturation (6). Note that, in this particular study, IVM was conducted using denuded GV oocytes collected after ovarian stimulation. The direct action of AMH on human oocyte maturation could be further investigated using COCs isolated from antral follicles of unstimulated ovarian tissue in patients with clearly documented menstrual cycle information.

Human GC culture was performed to test AMH effects on mural GCs derived from the preovulatory follicle. GC growth was restricted as indicated by the decreased cell confluency and cell numbers following rhAMH supplementation. Neither necroptosis nor apoptosis was induced by rhAMH treatment as suggested by the minimal phospho-MLKL signal and absence of cleaved caspases, respectively, in cultured GCs. There appeared to be a slight increase in the autophagy process, but a significant difference was not identified between the control and rhAMH-treated GCs. Therefore, the underlying biochemical pathways that led to limited GC growth *in vitro* remain to be evaluated in future studies. Nevertheless, the present data are consistent with and complement to observations in a previous *in vivo* study in nonhuman primates (3). When rhAMH was infused into macaque ovaries, mural GC division was inhibited in antral follicles as indicated by cell cycle arrest. Apoptosis was not identified in rhAMH-infused ovaries based on TUNEL staining. The rhAMH infusion induced beclin-1 protein expression in oocytes, suggesting an active autophagy process, but not in mural GCs of antral follicles. Taken together, AMH may inhibit mural GC proliferation without affecting cell viability during antral follicle maturation. In preovulatory follicles, decreased AMH production by CCs could be necessary for sustaining mural GC growth and oocyte viability.

In summary, the present *in vitro* studies demonstrate that reduced AMH action in COCs is beneficial for oocyte maturation during antral follicle development in primate species. CC-derived AMH is not essential for supporting oocyte developmental competence or mural GC viability. Thus, AMH expression levels in CCs may serve as a biomarker for oocyte maturation potential during IVF in patients. Effects of AMH depletion can be further studied in cultured COCs from patients in various age groups for the potential clinical applications in IVM or *in vitro* follicle maturation to improve oocyte maturation rates.

## Data availability statement

The original contributions presented in the study are included in the article/supplementary material. Further inquiries can be directed to the corresponding author.

## Ethics statement

The studies involving humans were approved by Institutional Review Board of Oregon Health &Science University and Ethics Committee of Ludwig-Maximilians-University in Munich. The studies were conducted in accordance with the local legislation and institutional requirements. Written informed consent for participation in this study was provided by the participants’ legal guardians/next of kin. The animal study was approved by Oregon National Primate Research Center Institutional Animal Care and Use Committee. The study was conducted in accordance with the local legislation and institutional requirements.

## Author contributions

FX: Visualization, Validation, Methodology, Investigation, Writing – review & editing, Writing – original draft, Formal analysis, Data curation. KB: Validation, Methodology, Writing – review & editing, Formal analysis, Data curation. NM: Resources, Methodology, Writing – review & editing, Data curation. SS: Visualization, Resources, Methodology, Writing – review & editing. AM: Supervision, Investigation, Writing – review & editing, Funding acquisition. DL: Supervision, Resources, Conceptualization, Writing – review & editing. TP: Funding acquisition, Supervision, Resources, Writing – review & editing. SM: Resources, Supervision, Writing – review & editing, Methodology. JX: Writing – review & editing, Writing – original draft, Validation, Supervision, Project administration, Investigation, Funding acquisition, Conceptualization.

## References

[1] Gruijters MJ Visser JA Durlinger AL Themmen AP. Anti-Mullerian hormone and its role in ovarian function. Mol Cell Endocrinol. (2003) 211:85–90. doi: 10.1016/j.mce.2003.09.024 14656480

[2] Xu J Xu F Letaw JH Park BS Searles RP Ferguson BM. Anti-Müllerian hormone is produced heterogeneously in primate preantral follicles and is a potential biomarker for follicle growth and oocyte maturation in *vitro* . J Assist Reprod Genet. (2016) 33:1665–75. doi: 10.1007/s10815-016-0804-3 PMC5234704 27638727

[3] Xu F Lawson MS Campbell SP Tkachenko OY Park BS Bishop CV. Stage-dependent actions of antimüllerian hormone in regulating granulosa cell proliferation and follicular function in the primate ovary. F S Sci. (2020) 1:161–71. doi: 10.1016/j.xfss.2020.10.005 PMC8329754 34355206

[4] Kedem A Hourvitz A Yung Y Shalev L Yerushalmi GM Kanety H. Anti-Müllerian hormone (AMH) downregulation in late antral stages is impaired in PCOS patients. A study in normo-ovulatory and PCOS patients undergoing in *vitro* maturation (IVM) treatments. Gynecol Endocrinol. (2013) 29:651–6. doi: 10.3109/09513590.2013.798279 23772776

[5] Kedem A Yung Y Yerushalmi GM Haas J Maman E Hanochi M. Anti Müllerian Hormone (AMH) level and expression in mural and cumulus cells in relation to age. J Ovarian Res. (2014) 7:113. doi: 10.1186/s13048-014-0113-3 25500128 PMC4269874

[6] Bedenk J Režen T Ramuta. TŽ Jančar N Bokal EV Geršak K. Recombinant anti-Müllerian hormone in the maturation medium improves the in *vitro* maturation of human immature (GV) oocytes after controlled ovarian hormonal stimulation. Reprod Biol Endocrinol. (2022) 20:18. doi: 10.1186/s12958-022-00895-5 35073905 PMC8785574

[7] Takahashi M Koide SS Donahoe PK. Müllerian inhibiting substance as oocyte meiosis inhibitor. Mol Cell Endocrinol. (1986) 47:225–34. doi: 10.1016/0303-7207(86)90116-4 2428679

[8] Yu X Li Z Zhao X Hua L Liu S He C. Anti-Müllerian hormone inhibits FSH-induced cumulus oocyte complex in *vitro* maturation and cumulus expansion in mice. Anim (Basel). (2022) 12:1209. doi: 10.3390/ani12091209 PMC9103408 35565634

[9] Zhang Y Shao L Xu Y Cui Y Liu J Chian RC. Effect of anti-Mullerian hormone in culture medium on quality of mouse oocytes matured in *vitro* . PLOS One. (2014) 9:e99393. doi: 10.1371/journal.pone.0099393 24932501 PMC4059625

[10] Tsafriri A Picard JY Josso N. Immunopurified anti-Müllerian hormone does not inhibit spontaneous resumption of meiosis in *vitro* of rat oocytes. Biol Reprod. (1988) 38:481–5. doi: 10.1095/biolreprod38.2.481 3358981

[11] Velásquez A Mellisho E Castro FO Rodríguez-Álvarez L. Effect of BMP15 and/or AMH during in *vitro* maturation of oocytes from involuntarily culled dairy cows. Mol Reprod Dev. (2019) 86:209–23. doi: 10.1002/mrd.23096 30548943

[12] Fasano G Dechène J Antonacci R Biramane J Vannin A Van Langendonckt A. Outcomes of immature oocytes collected from ovarian tissue for cryopreservation in adult and prepubertal patients. Reprod Biomed Online. (2017) 34:575–82. doi: 10.1016/j.rbmo.2017.03.007 28365199

[13] Xu J Lawson MS Mitalipov SM Park BS Xu F. Stage-specific modulation of antimüllerian hormone promotes primate follicular development and oocyte maturation in the matrix-free three-dimensional culture. Fertil Steril. (2018) 110:1162–72. doi: 10.1016/j.fertnstert.2018.07.006 PMC6226025 30396561

[14] Xu F Lawson MS Bean Y Ting AY Pejovic T De Geest K. Matrix-free 3D culture supports human follicular development from the unilaminar to the antral stage in *vitro* yielding morphologically normal metaphase II oocytes. Hum Reprod. (2021) 36:1326–38. doi: 10.1093/humrep/deab003 PMC8600176 33681988

[15] Bagnjuk K Stöckl JB Fröhlich T Arnold GJ Behr R Berg U. Necroptosis in primate luteolysis: a role for ceramide. Cell Death Discovery. (2019) 5:67. doi: 10.1038/s41420-019-0149-7 30774995 PMC6370808

[16] Peluffo MC Ting AY Zamah AM Conti M Stouffer RL Zelinski MB. Amphiregulin promotes the maturation of oocytes isolated from the small antral follicles of the rhesus macaque. Hum Reprod. (2012) 27:2430–7. doi: 10.1093/humrep/des158 PMC3398676 22593432

[17] Wolf DP Vandevoort CA Meyer-Haas GR Zelinski-Wooten MB Hess DL Baughman WL. *In vitro* fertilization and embryo transfer in the rhesus monkey. Biol Reprod. (1989) 41:335–46. doi: 10.1095/biolreprod41.2.335 2508776

[18] Pejovic T McCluggage WG Krieg AJ Xu F Lee DM Witkowski L. The dilemma of early preventive oophorectomy in familial small cell carcinoma of the ovary of hypercalcemic type. Gynecol Oncol Rep. (2019) 28:47–9. doi: 10.1016/j.gore.2019.02.002 PMC6402228 30886884

[19] Xu J Lawson MS Yeoman RR Pau KY Barrett SL Zelinski MB. Secondary follicle growth and oocyte maturation during encapsulated three-dimensional culture in rhesus monkeys: effects of gonadotrophins, oxygen and fetuin. Hum Reprod. (2011) 26:1061–72. doi: 10.1093/humrep/der049 PMC3079470 21362681

[20] Weenen C Laven JS Von Bergh AR Cranfield M Groome NP Visser JA. Anti-Mullerian hormone expression pattern in the human ovary: potential implications for initial and cyclic follicle recruitment. Mol Hum Reprod. (2004) 10:77–83. doi: 10.1093/molehr/gah015 14742691

[21] Sacchi S Marinaro F Xella S Marsella T Tagliasacchi D La Marca A. The anti-Mullerian hormone (AMH) induces forkhead box L2 (FOXL2) expression in primary culture of human granulosa cells in *vitro* . J Assist Reprod Genet. (2017) 34:1131–6. doi: 10.1007/s10815-017-0980-9 PMC5581793 28660501

[22] Chan LL Shen D Wilkinson AR Patton W Lai N Chan E. A novel image-based cytometry method for autophagy detection in living cells. Autophagy. (2012) 8:1371–82. doi: 10.4161/auto.21028 PMC3442883 22895056

[23] Ueno S Kuroda T Maclaughlin DT Ragin RC Manganaro TF Donahoe PK. Mullerian inhibiting substance in the adult rat ovary during various stages of the estrous cycle. Endocrinology. (1989) 125:1060–6. doi: 10.1210/endo-125-2-1060 2752965

[24] Baarends WM Uilenbroek JT Kramer P Hoogerbrugge JW van Leeuwen EC Themmen AP. Anti-müllerian hormone and anti-müllerian hormone type II receptor messenger ribonucleic acid expression in rat ovaries during postnatal development, the estrous cycle, and gonadotropin-induced follicle growth. Endocrinology. (1995) 136:4951–62. doi: 10.1210/endo.136.11.7588229 7588229

[25] Fasano G Moffa F Dechène J Englert Y Demeestere I. Vitrification of in *vitro* matured oocytes collected from antral follicles at the time of ovarian tissue cryopreservation. Reprod Biol Endocrinol. (2011) 9:150. doi: 10.1186/1477-7827-9-150 22112198 PMC3248844

[26] Abir R Ben-Aharon I Garor R Yaniv I Ash S Stemmer SM. Cryopreservation of in *vitro* matured oocytes in addition to ovarian tissue freezing for fertility preservation in paediatric female cancer patients before and after cancer therapy. Hum Reprod. (2016) 31:750–62. doi: 10.1093/humrep/dew007 26848188

[27] Grøndahl ML Nielsen ME Dal Canto MB Fadini R Rasmussen IA Westergaard LG. Anti-Müllerian hormone remains highly expressed in human cumulus cells during the final stages of folliculogenesis. Reprod Biomed Online. (2011) 22:389–98. doi: 10.1016/j.rbmo.2010.12.005 21353640

[28] Kedem-Dickman A Maman E Yung Y Yerushalmi GM Hemi R Hanochi M. Anti-Müllerian hormone is highly expressed and secreted from cumulus granulosa cells of stimulated preovulatory immature and atretic oocytes. Reprod Biomed Online. (2012) 24:540–6. doi: 10.1016/j.rbmo.2012.01.023 22421733

[29] Pavlić SD Milaković TT Horvat LP Čavlović K Vlašić H Manestar M. Genes for anti-Müllerian hormone and androgen receptor are underexpressed in human cumulus cells surrounding morphologically highly graded oocytes. SAGE Open Med. (2019) 7:2050312119865137. doi: 10.1177/2050312119865137 31360520 PMC6637837

